# Rift Valley fever seroprevalence and risk factors among human populations in Uganda’s cattle corridor: a cross-sectional study

**DOI:** 10.1136/bmjph-2025-002563

**Published:** 2025-07-31

**Authors:** Zacchaeus Anywaine, Daniel Wright, George M Warimwe, Pontiano Kaleebu, Alison Elliott, Christian Hansen

**Affiliations:** 1 Medical Research Council/Uganda Virus Research Institute, London School of Hygiene and Tropical Medicine Uganda Research Unit, Entebbe, Uganda; 2 Department of Clinical Research, London School of Hygiene and Tropical Medicine, London, UK; 3 Oxford Vaccine Group, Institute of Development and Regenerative Medicine, University of Oxford, Oxford, UK; 4 Centre for Tropical Medicine and Global Health, University of Oxford, Oxford, UK; 5 KEMRI-Wellcome Trust Research Programme, Kilifi, Kenya; 6 Uganda Virus Research Institute, Entebbe, Uganda; 7 MRC International Statistics & Epidemiology Group, London School of Hygiene and Tropical Medicine, London, UK; 8 Department of Infectious Disease Epidemiology and Prevention, Statens Serum Institut, Copenhagen, Denmark

**Keywords:** Humans, Cross-Sectional Studies, Age Factors, Prevalence, Zoonoses

## Abstract

**Introduction:**

Rift Valley fever (RVF) is an epidemic-prone zoonotic disease whose distribution of exposure is poorly described in endemic communities. We investigated the seroprevalence and risk factors for RVF among humans in Uganda’s cattle corridor.

**Methods:**

This cross-sectional study used data and stored plasma specimens collected by the Uganda population-based HIV impact assessment (UPHIA) survey 2016/2017. Participants were sampled from 35 districts of the cattle corridor. Ethical and regulatory approvals were obtained to use the UPHIA data and to test the specimens for the presence of RVF anti-Gn glycoprotein immunoglobulin G (anti-Gn IgG) antibodies using an enzyme linked immunosorbent Assay (ELISA). RVF seroprevalence was calculated in Stata with household as the random intercept, and associations between potential determinants and RVF seropositivity were assessed using mixed effects logistic regression analysis.

**Results:**

Overall, 267 households comprising 1319 individuals were included in the analysis. Over half (56.3%) of the participants were female, median age 22 (IQR 11–34) years. Most (84.2%) were rural and owned cattle (41.2%), sheep/goats (51.7%) and poultry (65%). The overall RVF seroprevalence was 10.24%, 95% CI 8.63% to 12.10%. RVF seropositivity was associated with advanced age (25–44 years adjusted OR 2.79 (95% CI 1.81 to 4.32); and 45–64 years (3.0 (95% CI 1.76 to 5.14)); ethnicity (Iteso 2.54 (95% CI 1.15 to 5.62), Langi 2.61 (95% CI 1.20 to 5.66) and Karamojong 3.70 (95% CI 1.61 to 8.47)); owning cattle (1.59 (95% CI 1.03 to 2.45)) and owning poultry (1.73 (95% CI 1.05 to 2.87)).

**Conclusions:**

RVF seropositivity is common among humans in Uganda’s cattle corridor and the risk of exposure is mostly determined by increasing age, ethnicity, owning cattle and poultry. Future RVF seroprevalence and risk evaluation studies should include poultry as potential determinants of infection.

**WHAT IS ALREADY KNOWN ON THIS TOPIC**

- Rift Valley fever (RVF) virus is a neglected category 4 pathogen whose exposure burden and risk factors are not fully studied in affected communities.

**WHAT THIS STUDY ADDS**

- This study shows that RVF is much common than would be expected from the number of reported cases during outbreaks, implying a significant incidence of asymptomatic infection or mild disease that remains undetected. The study shows that ethnicity, independent of spatial location and owning poultry are potential risk factors for RVF in humans.

**HOW THIS STUDY MIGHT AFFECT RESEARCH, PRACTICE OR POLICY**

- We present valuable data highlighting the RVF hotspot areas and populations with an increased risk of RVF in Uganda’s cattle corridor. This information provides insight on where vigilance in surveillance, public awareness campaigns, animal and human vaccination efforts when vaccines become available should be focused. Public access to these data will be valuable for inclusion in mathematical models of RVF disease dynamics and to support vaccine development. Future one-health RVF public health predictive, mitigation and control efforts shall, in addition to humans, animals and environment, consider including poultry as potential determinants of infection.

## Introduction

 Rift Valley fever (RVF) virus is an epidemic-prone pathogen neglected by most national disease control programmes and major global public health funders.^1^ The disease was first systematically documented in 1931,^2^ and over the years has caused outbreaks in Africa and the Arabian Peninsula.^3 4^ Outbreaks have occurred repeatedly in Kenya^5^ and Tanzania,^6^ yet Uganda has had an inter-outbreak span of close to 50 years.^7^ The sporadic outbreaks of RVF are associated with conditions that lead to flooding. Within Eastern Africa and the Arabian Peninsula, outbreaks are closely linked to the El Niño-Southern Oscillation phenomenon which causes heavy rains and flooding to the region.^8^ In other parts of Africa, outbreaks have occurred following flooding due to construction of hydroelectric power dams^9 10^ or large agriculture irrigation schemes.^11 12^ Aedes species mosquito eggs are the natural reservoirs of the virus and following flooding, hatch and transmit the virus to susceptible domestic and wild ungulates.^3 13^ Flooding also favours the mass breeding of Culex and other mosquito species which feed on infected animals and act as virus amplifying vectors to spread to other animals and humans.^14 15^ Humans acquire infection through contact with infected animal tissues mediated through nursing and assisting animal births, slaughtering and milking, consumption of unpasteurised milk and raw meat as well as sheltering with animals.^16 17^ In humans, a range of manifestations occurs, from asymptomatic and mild self-limiting influenza-like and gastrointestinal illness to life-threatening hepatitis, haemorrhage, encephalitis, renal failure or death, and miscarriage has been reported.^18^,^20^ Currently, there is no licensed vaccine for human use and many countries lack surveillance programmes, the cornerstone to timely outbreak control.

In Uganda, the virus was first isolated from mosquitoes in 1944,^21^ and the isolates were used to develop the livestock Smithburn vaccine.^22^ During this time, human accidental laboratory infections occurred and were reported.^23^ Human community outbreaks were reported in the 1960s^24^ after which cases were not reported until 2016.^7^ Between 2017 and 2020, several outbreaks occurred,^25^ with cases characterised by severe morbidity and high hospital-based mortality.^26^ A study in 2009^27^ in four central districts of Uganda found RVF antibodies in goats and concluded the virus was circulating in the country despite the absence of reported human and animal cases. Two studies have previously reported on RVF seroprevalence and risk factors among humans in Uganda; however, both were conducted during RVF outbreaks. One conducted in April 2016 and restricted to a single district had a seroprevalence of 12%^28^ whereas the other, undertaken between November 2017 and January 2018, had a seroprevalence of 29%.^29^ A comprehensive study on the extent to which humans have been previously exposed to RVF and associated risk factors in Uganda has never been conducted.

Livestock density is particularly high in the Uganda cattle corridor, a region comprised of about 35 districts,^30^ presenting a persistent risk to the resident human population. We hypothesised that the prevalence of previous exposure to RVF infection among humans in Uganda’s cattle corridor would be high. In this study, we investigated the prevalence of previous exposure to RVF by measuring plasma anti-RVF IgG antibodies among human residents in Uganda’s cattle corridor and assessed factors associated with RVF positivity.

## Materials and methods

### Study design and population

This cross-sectional study used epidemiological data and stored plasma samples from the Uganda population-based HIV impact assessment (UPHIA) survey 2016/2017 conducted by the Uganda Ministry of Health and partners.^31^ The UPHIA survey was conducted country-wide to assess HIV prevalence and antiretroviral treatment implementation in Uganda. It took place between August 2016 and March 2017 and offered household HIV counselling and testing to people aged 0–64 years. Blood specimens were collected and tested for HIV, hepatitis B and syphilis antibodies. Plasma specimens were stored at the Uganda Virus Research Institute (UVRI). The survey collected information from respondents on variables including age, sex, ethnicity, location (district, region and residence (rural or urban)), socioeconomic factors (such as education, religion, marital status and housing status), pregnancy status, environmental factors (including water source, mosquito net use and crowding (number of people per household)), and ownership of poultry, cattle, goats/sheep, dogs, donkeys and camels.

In this study, to investigate the seroprevalence and risk factors for RVF among humans within the cattle corridor, we tested stored plasma specimens to establish the presence of anti-RVF IgG antibodies. Data from laboratory tests were merged with data from the UPHIA survey questionnaires. The survey was conducted close to both the 2016 and 2017/2018 RVF outbreaks in Uganda and included people aged between 0 and 64 years.

### Study area

The cattle corridor occupies about 35% of Uganda’s land surface area and stretches from the North-East to the South-West. At the time of designing the study, there were 112 districts in Uganda, out of which 35 made up the cattle corridor. Between 2017 and 2020, 95% of the 52 RVF confirmed cases by the Uganda National Haemorrhagic Fever surveillance programme were residents within the cattle corridor districts and livestock contact was the main risk factor.^25^ We hypothesised that a high proportion of the human population in the cattle corridor was exposed to RVF than known through the national surveillance programme. The area is characterised by a semiarid climate, unreliable rainfall patterns, droughts and flooding during seasonal rains. The vegetation is savannah grassland with shrubs, interspersed with mostly seasonal rivers, some permanent rivers and lakes located distant from each other and prone to periodic flooding. There are two national game parks (Lake Mburo and Kidepo Valley), four game reserves (Ziwa rhino sanctuary, Matheniko, Bokora and Pian Upe game reserves), and the corridor borders Murchison Falls national park. These diverse environmental and geographical features favour ecological multispecies coexistence involving humans, animals and RVF-transmitting mosquito vectors. The cattle corridor is famous for livestock farming and the 2008 livestock census by the Uganda Bureau of Statistics and the Ministry of Agriculture, Animal Industry and Fisheries reported the area to hold 54% of the country’s cattle, 41% of goats and 66% of sheep,^32^ yet only 23.4% (8.1 million) of the total human population for Uganda.^33^

### Sampling for the UPHIA survey

The UPHIA survey sampling frame comprised all households in the country based on the enumeration areas (districts) of the 2014 National Housing and Population census.^33^ Households were sampled from districts in proportion to total households in the country. All individuals in selected households were eligible for enrolment into the survey.

### Sample size estimation for RVF seroprevalence

A minimum sample size of 1414 respondents from 472 households was required based on the estimated household size of 3 persons per household from the UPHIA survey. The sample size for this clustered survey was calculated using the formula n=d($Z^{2}p\frac{\left(1-p\right)}{e^{2}}$),^34^ applied with design effect (d)=2, critical value at the 95% confidence level (Z)=1.96, hypothesised prevalence (p)=0.08 and required precision (e)=±25% of p, or e=0.02. The sample size was calculated based on the 12% RVF prevalence during the 2016 outbreak in Kabale district in South-Western Uganda,^28^ and we predicted a lower prevalence (p=8% or 0.08) across the cattle corridor since the UPHIA survey was conducted during an interepidemic period. The rationale for the clustered survey design was to maximise the opportunity to investigate the prevalence of RVF in Uganda as well as the relationship between crowding at household level represented by household size as a proxy for potential human-to-human transmission and RVF seropositivity.

### Sampling for RVF seroprevalence study from the UPHIA survey

The RVF seroprevalence study maintained the UPHIA survey cluster sampling design. A cluster was a household, defined by the UPHIA survey as ‘a person or group of persons related or unrelated to each other who live in the same compound (fenced or unfenced), share the same cooking arrangements, and have one person whom they identify as head of that household’.^31^ The RVF seroprevalence study sampling frame was the list of households that participated in the UPHIA survey and located within the 35 districts of Uganda’s cattle corridor. In the UPHIA survey, 96.9% (12,483) of 12 882 eligible households in the country participated in the survey, of which 3920 were located in the cattle corridor. Using simple random sampling without replacement, 472 households (n=1581 individuals) were selected.

### Laboratory procedures

RVF IgG antibodies were measured using ELISA as previously described.^35^ Pooled RVF-positive sera from RVF-confirmed survivors from Uganda were the positive control. Briefly, flat bottom 96 well plates were coated with 50 µL per well of Gn antigen at 1 µg/mL diluted in phosphate buffered saline (PBS) and incubated overnight at 4°C. All incubation steps hereafter were at room temperature. The following day, plates were washed 6 times with 1xPBS/0.05% tween 20 (PBS/T) wash buffer and tapped dry. Plates were blocked with 100 µL per well of casein (1% casein in PBS) and incubated for 1 hour. Blocking solution was discarded, plates tapped dry and Test sera, internal control serum and negative control were diluted in casein and added in duplicate to the plate (50 µL/well). Serial dilutions (1:2) of the positive control (used to generate a standard curve) were made in duplicate starting with 1:100 down to 1: 51 200 and added to the plate (50 µL/well). Sera were incubated for 2 hours. Plates were washed and 50 µL per well of goat anti-human IgG-alkaline phosphatase secondary antibody (at 1:1000 in casein) was added, incubated for 1 hour and washed. p-nitrophenylphosphate substrate (50 µL/well) was added, and colour reaction left to develop at room temperature in the dark. Optical densities (ODs) were read at 405 nm on the BioTech microplate reader. ELISA units (AU) of each serum sample were calculated by extrapolation from the standard curve. Serum specimens scoring an OD value greater than the mean+2 SD of the negative control were deemed positive. Each sample was run in duplicate, and OD values were averaged. Any sample with OD >2.5 or duplicate wells with OD coefficient of variation >20% was excluded from this analysis as repeat testing was not done.

### Study variables

**Figure 1:**
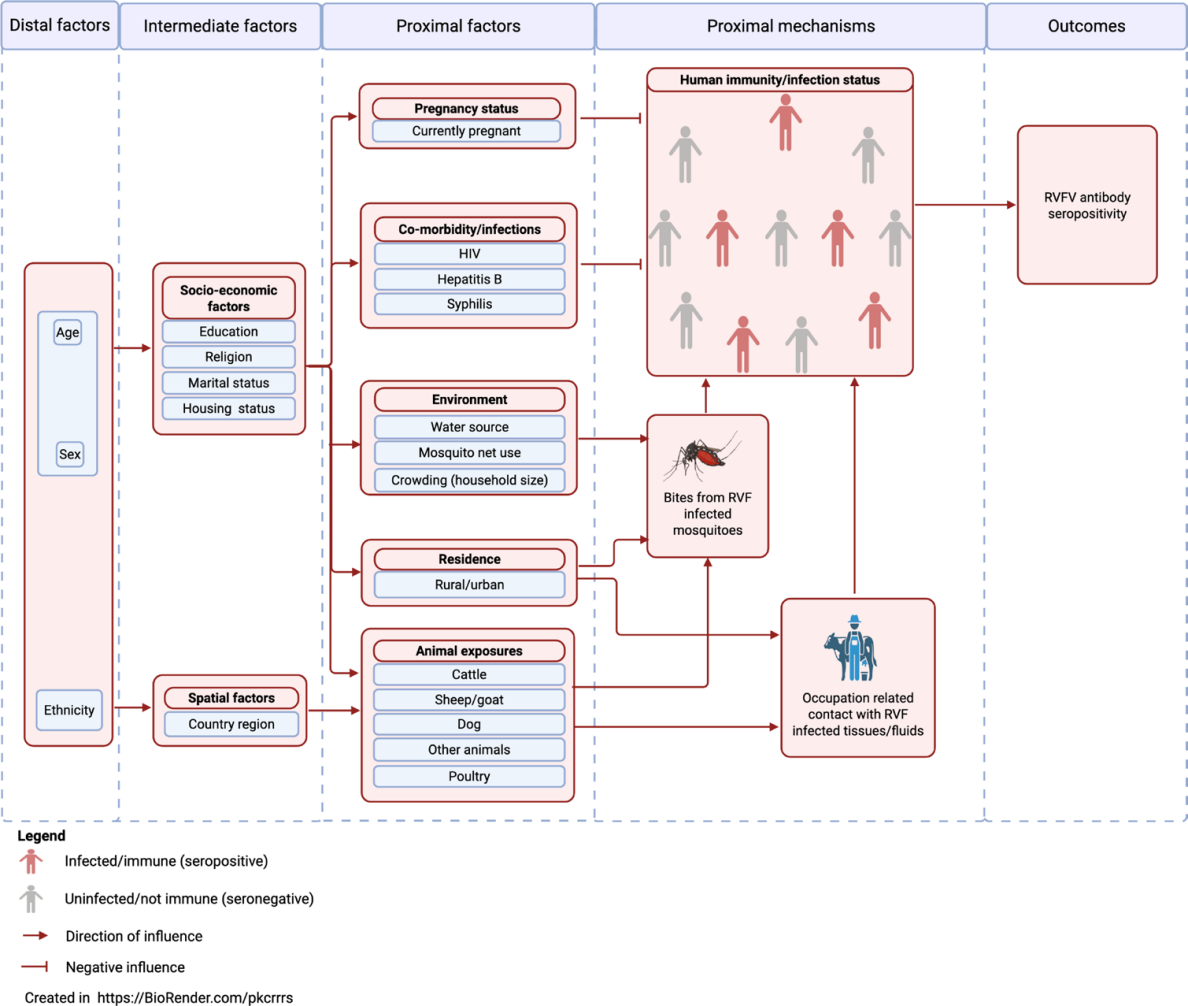
Conceptual framework to investigate the determinants of RVF seropositivity among human populations in Uganda’s cattle corridor districts. RVF, Rift Valley fever; RVFV, RVF virus.

RVF seropositivity defined as presence of anti-RVF-Gn IgG antibodies using ELISA provided our binary outcome variable. We developed a conceptual framework to map parallel and hierarchical relationships among explanatory variables and their relationship with the outcome (figure 1) as previously described with slight modification.^36^,^38^ We identified plausible biological and literature-established sociodemographic, economic and environmental connections and stratified variables hierarchically as distal, intermediate, and proximal factors. The presence or increase in level of a factor, for example, age (distal factor) influences one’s marital status (intermediate factor), which in turn influences one’s HIV serostatus (proximal factor). HIV positive status negatively influences (arrow with blunted-end head) one’s immunity. A suppressed immunity increases the chances of RVF infection/overall RVF seroprevalence. Most variables were recoded for this RVF seroprevalence analysis as indicated in the variables code sheet (online supplemental Table 1).

### Statistical analysis

Data from the UPHIA survey database were merged with the ELISA RVF seropositivity results (outcome). Participants from the UPHIA database without samples tested (no outcome) were excluded from this analysis and reasons for exclusion captured. The proportion of individuals with previous RVF exposure was calculated in STATA V.18.5 (Stata), with household as the random intercept to capture clustering at the household level. The analysis was based on the conceptual framework (figure 1) developed a priori for the relationship between variables. The proposed conceptual causal pathway was considered in determining the order in which variables were adjusted. Since the direction of the causal pathway starts with distal variables through intermediate to proximal and then outcome, distal variables were only adjusted for each other, intermediate for each other and distal, whereas proximal for each other, for intermediate and distal covariates. Practically, the associations between potential determinants and RVF seropositivity were assessed in three stages. In the first stage (level 1), a univariable mixed effects logistic regression analysis was done to determine the association between each individual parameter and RVF seropositivity. This provided unadjusted ORs with 95% CIs. Variables significant at p≤0.1 were selected for fitting the final stage (level 3) analysis models. Multicollinearity and correlation were tested for all predictor variables selected for level 3 using the variable inflation factor (VIF) and correlation, respectively. We aimed for a non-existence of multicollinearity if the VIF was below 5.0, and correlation coefficient between −0.5 and +0.5. At the second (level 2) and third stages (level 3), multivariable mixed effects logistic regression models were fitted. Associations between predictor variables and RVF seropositivity were assessed using adjusted ORs (aOR) and 95% CIs. At level 2, each variable was independently adjusted for more distal determinants (including age and sex selected for inclusion a priori) found to be statistically significant (p≤0.05). At the final stage (level 3), three models were fitted. These included model 1 (incorporating only distal variables), model 2 (for intermediate and distal variables) and model 3 (proximal variables adjusted for intermediate and distal variables). Variables were selected for inclusion into the level 3 model-based analysis if found statistically significant at p≤0.1 in level 1 and/or level 2 analysis. For each of models 1, 2 and 3, variables were adjusted for each other and for the statistically significant (p≤0.05) distal determinants including age and sex planned a priori. An interaction analysis to investigate joint effects and effect modification was undertaken for all independent predictors established from the three level 3 models using the interaction parameter p value from the likelihood ratio test. A coefficients plot was drawn using GraphPad Prism V.10.2.1 (339) to present variables included in the three predictive models (models 1, 2 and 3) of RVF seropositivity following regression analysis at level 3.

### Patient and public involvement

The survey participants were not directly involved in this research as we analysed archived data and stored plasma specimens. However, prior to study initiation, the proposal was presented to the Medical Research Council/Uganda Virus Research Institute and London School of Hygiene and Tropical Medicin Uganda Research Unit community advisory board which comprised representatives of medical personnel, research participants and the political, media, religious and lay communities. The board was supportive of the aims of the study.

## Results

### Characteristics of participants

**Figure 2:**
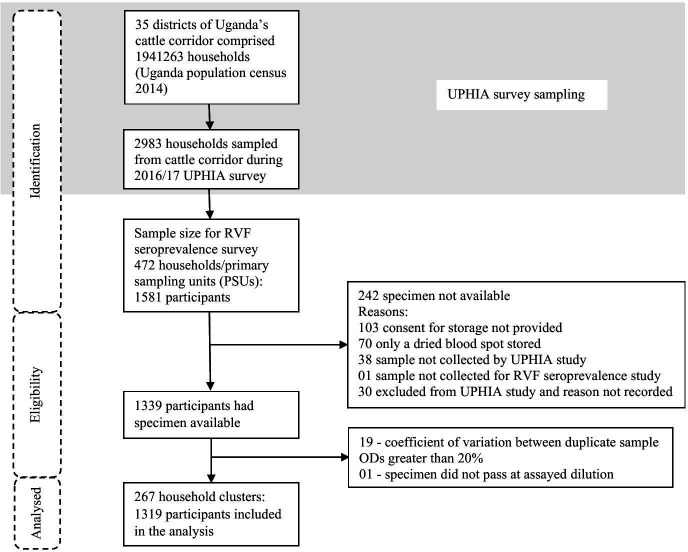
Study schema indicating the number of participants included at each stage of the study. ODs, optical densities; RVF, Rift Valley fever; UPHIA, Uganda population-based HIV impact assessment.

The UPHIA survey sampled 2983 households from the 35 cattle corridor districts in Uganda, of which 472 households (1581 participants) were sampled for the RVF seroprevalence survey (figure 2). 242 participants had no sample stored and were excluded, while the ELISA-IgG test failed quality control for 20 individuals who were also excluded.

267 households (1319 individuals) were included in this analysis, out of whom 56.3% were females and 43.7% males, median age 22 (IQR 11–34) years (table 1). 38% of participants were from the North-Eastern region, 22.5% from mid-North and 18.8% from Central region 2. Most (84.4%) were rural, and of different ethnicities mainly comprising the Langi (22.4%), Iteso (20.0%), Baganda (15.6%), Banyankore (14.9%) and Karimojong (10.8%). Forty-one percent (41.2%) owned cattle, 51.7% sheep/goats, 21.7% dogs, 65% poultry and 78.2% a mosquito net.

**Table 1:** Characteristics of the population and RVF seroprevalence among humans in Uganda’s cattle corridor

Variable	Distribution of the study population N (%)	Number of samples positive for RVF (prevalence) N (%)
Distal variables		
Age group (MeSH age category)		
0–24 years	654 (49.6)	38 (5.8)
25–44 years	468 (35.5)	68 (14.5)
45–64 years	197 (14.9)	29 (14.7)
Age (years as continuous variable)		
Mean (SD)	24 (16) years	
Median, IQR	22 (11–34) years	
Sex		
Female	743 (56.3)	67 (9.0)
Male	576 (43.7)	68 (11.8)
Ethnicity		
Banyankore	196 (14.9)	10 (5.1)
Baganda	206 (15.6)	16 (7.8)
Iteso	264 (20.0)	29 (11.0)
Langi	295 (22.4)	33 (11.2)
Karamojong	143 (10.8)	23 (16.1)
Other ethnicity	215 (16.3)	24 (11.2)
Intermediate variables		
Highest level of education attained		
No formal education	155 (14.3)	24 (15.5)
Primary (P1–P7)	615 (56.6)	82 (13.3)
Secondary (S1–S6)	248 (22.8)	19 (7.7)
Tertiary (more than secondary)	69 (6.4)	5 (7.3)
Religion		
Catholic	533 (48.8)	59 (11.1)
Protestant/Anglican	325 (29.8)	40 (12.3)
Muslim	75 (6.9)	7 (9.3)
Other religions	159 (14.6)	23 (14.5)
Marital status		
Never married	315 (28.9)	28 (8.9)
Married or living together	637 (58.3)	85 (13.3)
Separated (divorced/widowed)	140 (12.8)	17 (12.1)
Housing status (house roof material)		
Corrugated iron (mabati)	720 (54.6)	58 (8.1)
Thatch/palm leaf (makuti)	573 (43.4)	74 (12.9)
Other roof type	26 (2.0)	3 (11.5)
Region of the country		
Central 1	96 (7.3)	13 (13.5)
Central 2	248 (18.8)	19 (7.7)
Mid-North	297 (22.5)	35 (11.8)
North-Eastern	503 (38.1)	57 (11.3)
South-Western	175 (13.3)	11 (6.3)
Proximal variables		
Currently pregnant		
No	552 (89.3)	60 (10.9)
Yes	58 (9.7)	3 (5.2)
HIV status		
Negative	1254 (95.1)	129 (10.3)
Positive	65 (4.9)	6 (9.2)
Hepatitis B exposure		
Negative	1245 (94.4)	123 (9.9)
Positive	74 (5.6)	12 (16.2)
Syphilis exposure		
Negative	1017 (93.0)	118 (11.6)
Positive	77 (7.0)	12 (15.6)
Water source		
Underground/borehole	680 (51.6)	82 (12.1)
Piped water	166 (12.6)	12 (7.2)
Surface water	442 (33.5)	36 (8.1)
Other water sources	31 (2.4)	5 (16.1)
Mosquito net use		
No	287 (21.8)	26 (9.1)
Yes	1032 (78.2)	109 (10.6)
Crowding (household/cluster size)		
1–3	180 (13.7)	20 (11.1)
4–6	435 (33.0)	42 (9.7)
7–9	411 (31.2)	39 (9.5)
10 or more	293 (22.2)	34 (11.6)
Residence		
Urban	206 (15.6)	13 (6.3)
Rural	1113 (84.4)	122 (11.0)
Own cattle		
No	776 (58.8)	59 (7.6)
Yes	543 (41.2)	76 (14.0)
Own sheep/goat		
No	637 (48.3)	52 (8.2)
Yes	682 (51.7)	83 (12.2)
Own dog		
No	1033 (78.3)	98 (9.5)
Yes	286 (21.7)	37 (12.9)
Own poultry		
No	462 (35.0)	29 (6.3)
Yes	857 (65.0)	106 (12.4)
Own other animals (camel, horse, donkey)		
No	1192 (90.4)	120 (10.1)
Yes	127 (9.6)	15 (11.8)

MeSH, medical subject heading; RVF, Rift Valley fever.

### RVF seroprevalence

**Figure 3:**
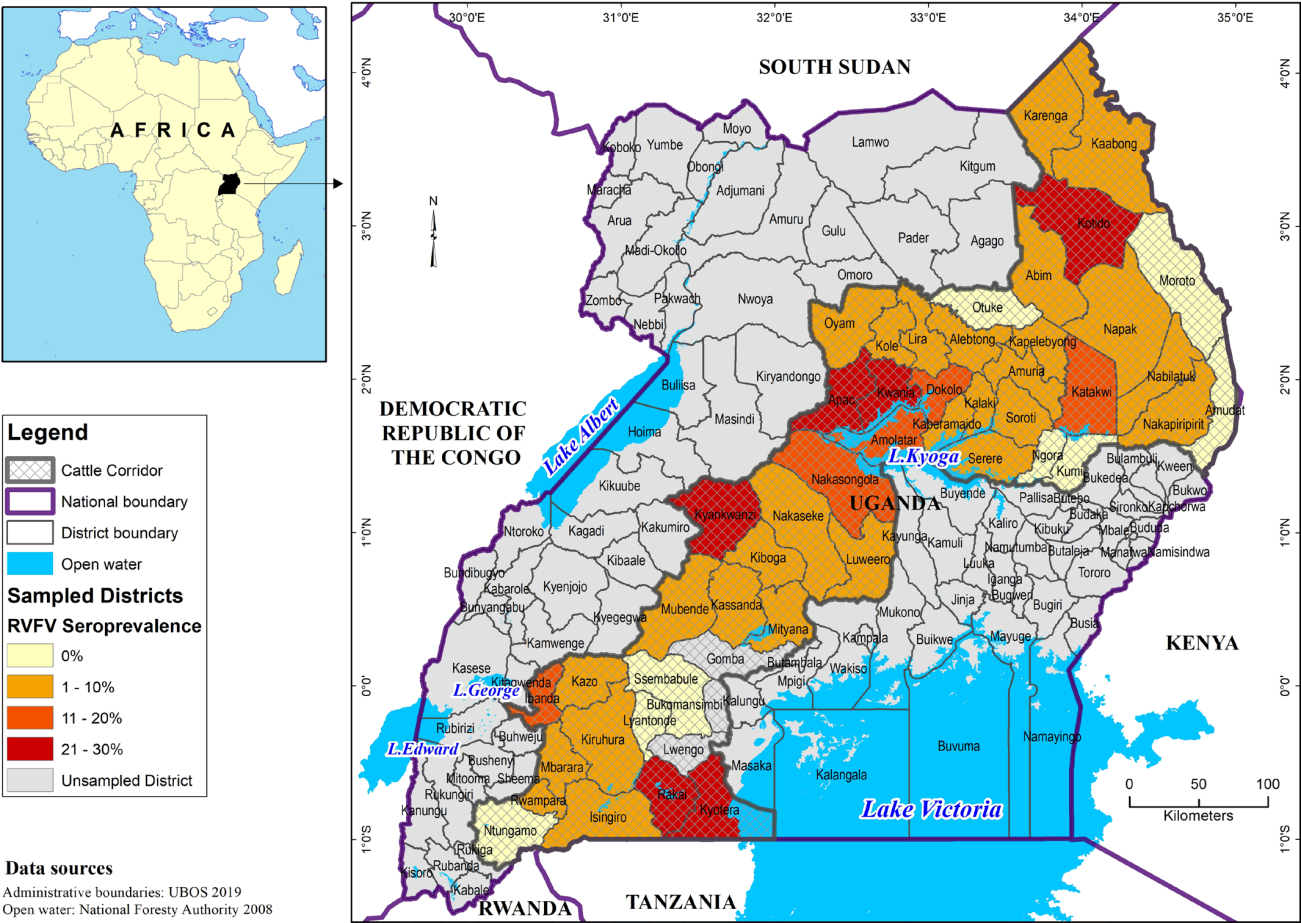
Map of Uganda showing the prevalence of RVF antibodies among humans in each sampled cattle corridor district. RVF, Rift Valley fever; UBOS, Uganda Bureau of Statistics.

The overall RVF seroprevalence was 10.24%, 95% CI 8.63% to 12.10%, equivalent to 10 240 people exposed per 100 000 in the population, and the median antibody levels were 51.4 (range 28.85–1773) AU/mL. The prevalence was generally higher in males and those aged 45–64 years and 25–44 years than females and 0–24 years, respectively (table 1), although also non-significantly higher for females than males in the age groups 6–18 years (online supplemental figure 1). The prevalence was high in rural than urban areas, central region 1 occupied mainly by Baganda, mid-Northern region occupied by Langi, North-Eastern region occupied by Iteso and Karimojong, and least in South-Western region among the Banyankore. RVF seroprevalence was highest among households owning cattle, dogs, poultry, and sheep/goats. The prevalence of RVF antibodies among humans in each sampled cattle corridor district is indicated in figure 3.

### RVF risk factors

#### Univariable and multivariable analysis for the predictors of RVF seropositivity

Age, sex, ethnicity, education, region of the country, housing status, water source, residence, owning cattle, sheep/goat and poultry had a p≤0.1 on univariable (level 1) and/or multivariable (level 2) analysis and were selected for the final (level 3) multivariable regression analysis. On testing the selected variables for multicollinearity and correlation (online supplemental Tables 2,3), respectively, all variables had VIF<5.0 (mean VIF 1.12) and correlation coefficient between −0.5 and +0.5, thus neither was found and all were considered for the final multivariable analysis. Statistical significance was established for the final multivariable regression models when variables achieved a p≤0.05 as shown in table 2.

**Table 2:** Factors associated with RVF seropositivity among 1319 individuals assessed from Uganda’s cattle corridor

Variable	Univariable mixed effects logistic regression analysis		Multivariable mixed effects logistic regression analysis				
	Level 1: Unadjusted OR, (95% CI)	Overall p value	Level 2: Each variable adjusted individually for statistically significant (at 5% level) distal factors only, aOR (95% CI)	P value	Level 3: Variables in each final model category adjusted for each other and for statistically significant (at 5% level) distal factors, aOR (95% CI)	P value	Final model category
Distal variables							
Age group (Medical Subject Heading (MeSH) age category)							
0–24 years	Reference (Ref)	<0.001	–	–	Ref	<0.001	Model 1*
25–44 years	2.82 (1.83 to 4.35)		–		2.79 (1.81 to 4.32)		
45–64 years	2.92 (1.71 to 4.98)		–		3.00 (1.76 to 5.14)		
Sex							
Female	Ref	0.081	–	–	Ref	0.055	Model 1*
Male	1.39 (0.96 to 2.02)		–		1.45 (0.99 to 2.11)		
Ethnicity							
Banyankore	Ref	0.043	–	–	Ref	0.050	Model 1*
Baganda	1.62 (0.69 to 3.79)		–		1.79 (0.76 to 4.20)		
Iteso	2.48 (1.12 to 5.46)		–		2.54 (1.15 to 5.62)		
Langi	2.38 (1.10 to 5.12)		–		2.61 (1.20 to 5.66)		
Karamojong	3.67 (1.61 to 8.35)		–		3.70 (1.61 to 8.47)		
Other ethnicity	2.46 (1.11 to 5.49)		–		2.61 (1.16 to 5.84)		
Intermediate variables							
Highest level of education attained							
No formal education	Ref	0.053	Ref	0.139	Ref	0.250	Model 2**†**
Primary (P1–P7)	0.86 (0.51 to 1.45)		1.09 (0.58 to 2.06)		1.18 (0.62 to 2.22)		
Secondary (S1–S6)	0.46 (0.24 to 0.90)		0.65 (0.29 to 1.44)		0.75 (0.34 to 1.67)		
Tertiary	0.43 (0.15 to 1.24)		0.47 (0.15 to 1.46)		0.55 (0.17 to 1.76)		
Religion							
Catholic	Ref	0.594	Ref	0.362	–	–	–
Protestant/Anglican	1.16 (0.73 to 1.83)		1.41 (0.86 to 2.29)		–		
Muslim	0.84 (0.35 to 2.02)		1.14 (0.44 to 2.95)		–		
Other religions	1.41 (0.80 to 2.47)		1.62 (0.88 to 2.95)		–		
Marital status							
Never married	Ref	0.116	Ref	0.871	–	–	–
Married/living together	1.66 (1.03 to 2.67)		0.92 (0.48 to 1.77)		–		
Separated (divorced/widowed)	1.46 (0.75 to 2.87)		0.81 (0.35 to 1.87)		–		
Housing status (house roof material)							
Corrugated iron	Ref	0.025	Ref	0.452	Ref	0.649	Model 2**†**
Thatch/palm leaf	1.72 (1.16 to 2.54)		1.39 (0.83 to 2.31)		1.26 (0.72 to 2.20)		
Other roof type	1.52 (0.40 to 5.74)		1.21 (0.31 to 4.70)		0.75 (0.14 to 3.89)		
Region of the country							
Central 1	Ref	0.155	Ref	0.079	Ref	0.099	Model 2**†**
Central 2	0.52 (0.23 to 1.17)		0.39 (0.17 to 0.91)		0.42 (0.18 to 1.00)		
North-East	0.81 (0.40 to 1.64)		0.24 (0.07 to 0.76)		0.26 (0.08 to 0.84)		
Mid-North	0.82 (0.39 to 1.74)		0.88 (0.19 to 4.10)		1.12 (0.21 to 5.98)		
South-Western	0.41 (0.17 to 1.02)		0.63 (0.23 to 1.77)		0.52 (0.16 to 1.61)		
Proximal variables							
Currently pregnant							
No	Ref	0.182	Ref	0.189	–	–	–
Yes	0.43 (0.13 to 1.48)		0.43 (0.12 to 1.52)		–		
HIV status							
Negative	Ref	0.784	Ref	0.441	–	–	–
Positive	0.88 (0.36 to 2.16)		0.70 (0.28 to 1.75)		–		
Hepatitis B exposure							
Negative	Ref	0.117	Ref	0.499	–	–	–
Positive	1.73 (0.87 to 3.42)		1.28 (0.63 to 2.60)		–		
Ever had syphilis infection							
Negative	Ref	0.358	Ref	0.813	–	–	–
Positive	1.38 (0.69 to 2.74)		1.09 (0.54 to 2.20)		–		
Water source							
Underground/borehole	Ref	0.082	Ref	0.173	Ref	0.202	Model 3**‡**
Piped water	0.54 (0.28 to 1.07)		0.77 (0.37 to 1.60)		1.44 (0.64 to 3.23)		
Surface water	0.64 (0.41 to 0.99)		0.78 (0.48 to 1.27)		0.77 (0.48 to 1.23)		
Other water sources	1.36 (0.46 to 4.02)		2.58 (0.81 to 8.29)		2.07 (0.66 to 6.47)		
Mosquito net use							
No	Ref	0.474	Ref	0.284	–	–	–
Yes	1.20 (0.73 to 1.95)		1.32 (0.80 to 2.17)		–		
Crowding (household/cluster size)							
1–3	Ref	0.765	Ref	0.792	–	–	–
4–6	0.86 (0.47 to 1.56)		0.91 (0.49 to 1.66)		–		
7–9	0.83 (0.45 to 1.54)		0.84 (0.45 to 1.57)		–		
ten or more	1.08 (0.57 to 2.05)		1.10 (0.57 to 2.12)		–		
Residence							
Urban	Ref	0.049	Ref	0.067	Ref	0.076	Model 3**‡**
Rural	1.89 (1.00 to 3.55)		1.83 (0.95 to 3.49)		1.92 (0.93 to 3.92)		
Own cattle							
No	Ref	<0.001	Ref	0.002	Ref	0.034	Model 3**‡**
Yes	2.01 (1.37 to 2.95)		1.92 (1.27 to 2.91)		1.59 (1.03 to 2.45)		
Own sheep/goat							
No	Ref	0.024	Ref	0.033	Ref	0.786	Model 3**‡**
Yes	1.57 (1.06 to 2.32)		1.55 (1.04 to 2.32)		1.06 (0.68 to 1.65)		
Own dog							
No	Ref	0.117	Ref	0.189	–	–	–
Yes	1.43 (0.92 to 2.22)		1.35 (0.86 to 2.12)		–		
Own poultry							
No	Ref	0.001	Ref	0.002	Ref	0.033	Model 3**‡**
Yes	2.15 (1.37 to 3.38)		2.11 (1.32 to 3.37)		1.73 (1.05 to 2.87)		
Own other animals (camel, horse, donkey)							
No	Ref	0.563	Ref	0.735	–	–	–
Yes	1.20 (0.64 to 2.26)		1.12 (0.58 to 2.17)		–		

* Model only including distal covariates.

† Model including intermediate covariates adjusting for distal.

‡ Model including proximal covariates adjusting for distal.

aOR, adjusted OR; RVF, Rift Valley fever.

At both univariable and multivariable analysis, the odds of testing positive for RVF were two to three times higher among people aged 25–44 and 45–64 years compared with those much younger (0–24 years). Similarly, compared with the Banyankore, the risk nearly doubled among Baganda, was two and a half times higher among the Iteso, Langi and other tribes, and nearly quadrupled among the Karimojong. Other exposures associated with a statistically significant risk of RVF seropositivity at both univariable (level 1) and multivariable levels (level 2) included owning cattle and poultry. Among those owning cattle and poultry, the odds were nearly twice on univariable analysis but reduced on multivariable level 3 analysis to values of equal relevance. Sex was associated with a borderline significant (p=0.055) 45% increase in the odds of RVF seropositivity following adjustment for age and ethnicity. The North-Eastern region of the country was the only factor associated with a significant reduction in the odds (aOR 0.26; 95% CI 0.08 to 0.84) of RVF seropositivity compared with Central region 1 as the ORs are wholly below 1. The likelihood ratio test p values for the pairwise testing of interaction among variables independently predictive of RVF seropositivity (age/ethnicity, age/own cattle, age/own poultry, ethnicity/own cattle, ethnicity/own poultry, own cattle/own poultry) were not statistically significant at a 5% level as indicated in online supplemental Table 4). Other variables that showed associations in univariable analyses (such as education, wealth represented by housing status, rural residence, water source and owning sheep/goat) were no longer significant following adjustment for age, sex and ethnicity in the final level 3 model-based analysis. A summary of the variables included in the final regression analysis models (models 1, 2 and 3) and those established to be associated with RVF seroprevalence among humans in this population is indicated in online supplemental figure 2.

## Discussion

In this study, we investigated the seroprevalence and risk factors for RVF among human populations in Uganda’s cattle corridor. The prevalence of RVF antibodies was common, and risk factors such as owning cattle and poultry as well as demographics such as age and ethnicity were independent predictors of RVF previous exposure among humans within this region.

The result encompasses our hypothesised seroprevalence value of 8.0% measured with a precision of ±25%. We hypothesised a somewhat lower value compared with the 12% seroprevalence measured in humans during the outbreak in Kabale district in 2016^28^ because our samples were collected in the subsequent interepidemic period. However, a systematic review by Clark *et al*^39^ indicated that RVF seroprevalence may not differ greatly between outbreak and interepidemic periods and the upper limit of our CI accords with this. Perhaps surprisingly, our 95% CIs also encompass the 11.5% RVF prevalence among abattoir workers sampled from four cities (Lira, Soroti, Mbale and Kampala) in Uganda—a group considered at higher risk than the general population. Meanwhile, a comparison of our results to those of recent studies shows that RVF seroprevalence is highly heterogeneous. The seroprevalence obtained in our study is within the range observed among pastoralists in Ethiopia,^40^ higher than observed in South Africa^41^ and Senegal,^42^ and yet much lower than a study in Chad.^43^ Together, these findings highlight an important fact that the exposure burden of RVF is varied; thus, there should be collective vigilance and effort to develop effective interventions for all populations.

Until recently, since the 1960s, human RVF outbreaks have been infrequently reported in Uganda. In 2016, three non-fatal human cases were reported in the South-Western district of Kabale.^28^ Presumably, the virus was circulating undetected in humans with mild or asymptomatic presentation, or diagnosed as other endemic tropical infections due to the broadly shared symptomatology. This hypothesis is supported by the seropositivity identified in goats in 2010^27^ and by the moderate seroprevalence in humans that we have observed. In South Africa, a study established that RVF transmissions occur in humans despite the absence of apparent clinical disease.^41^ Therefore, in the Ugandan context, the long interoutbreak span was probably not because of absence of disease, or only mild cases occurring, but also because of limited surveillance, awareness and interest for nearly half a century.

In our study, occupational exposures such as owning cattle and poultry, and demographics including age and ethnicity were independent predictors of RVF seropositivity among humans in Uganda. Demographic, animal-related and environmental factors are known to influence the risk of acquiring RVF in humans. A systematic review and meta-analysis that appraised 16 studies spanning 24 years with a total sample size of 15 069 participants and 1322 laboratory confirmed RVF cases reported that RVF seropositivity is associated with male sex and age; contact with aborted animal tissues, birthing, slaughtering, skinning, milking and drinking raw milk; and sheltering with animals and home flooding.^16^ Different animal species have varied susceptibility to RVF and species-specific risk quantification to humans has not been studied to aid changes in animal husbandry practices that could in part mitigate this zoonosis. A countrywide animal study from Uganda in 2017 found the prevalence of RVF antibodies to be fourfold higher in cattle (10.7%) than goats (2.6%) and sheep (2.0%) and differed among these animal species by region.^44^ There is currently little data as to which of the three most herded livestock is a major risk factor for humans. Sheep and goats succumb most to infection, and this could be related to their high turnover that presents frequent susceptibles within the herds. However, animal challenge models have shown species differences in the duration of viraemia and viral loads following infection^45^ with sheep exhibiting higher viral loads (10^5^–10^6^ pfu/mL lasting 1–9 days) than goats and cattle (10^3^–10^6^ pfu/mL for 1–4 days). These are major determinants of transmissibility. We are unable to delineate the reasons for the high risk associated with owning cattle in this study, and we believe more human–animal risk correlation studies are warranted. These studies could be vital in answering questions important in focusing predictive efforts such as the choice of animal species for sentinel herd surveillance or mitigation measures related to vaccine rationalisation in case of scarcity, and emergencies such as bioterrorism or accidental introduction of the virus in naïve ecologies.

The association between owning poultry and increased odds of RVF seropositivity was unexpected. Birds tend to be resistant to RVF infection.^9^ Earlier studies of RVF in Uganda in 146 birds belonging to 34 bird species did not find RVF antibodies or isolate the virus in birds.^24 46^ RVF experimental infection of the Sudan dioch (Quelea quelea aethiopica) in Kenya failed to establish viraemia or elicit neutralising antibodies.^47^ One possible theory proposed by Gerdess and not studied further in relation to the role of birds in RVF transmission was the possibility of mosquito eggs dispersion by wading birds.^9^ One study in Uganda reported that some mosquito species such as Culex and Mansonia (Coquillettidia), which are currently known to transmit RVF, prefer biting domestic fowl.^48^ It is possible that unmeasured confounders could have contributed to the observed relationship. However, in most rural communities similar to the population analysed in this study, domestic fowl share shelter and stay closest to humans compared with other animals. We infer that this close interaction could predispose humans to increased mosquito bites and subsequently mosquito-transmitted infections.

We found age and ethnicity but not sex to be predictors of RVF seropositivity in humans in a multivariable regression analysis. The association between age and RVF seropositivity was consistent with the results of two systematic reviews^16 39^ where 7 and 11 studies reported a positive association, respectively. In our study, the risk for RVF seropositivity increased with age, but data were not collected for adults beyond 64 years in the parent UPHIA study. One study in Kenya found the risk of RVF previous exposure to be highest among people over 65 years.^49^ This age-associated increase is likely due to cumulative life-time exposure rather than increased risk of acute RVF infections. Longitudinal studies among human survivors have shown antibodies lasting over 10 years.^35 50^ We found a weak association between male sex and RVF seropositivity, thus adding to the controversy found by Clark *et al* where in three studies the risk was increased in males, in two studies there was no association, and in one study, it was reduced compared with females.^39^ The results from these studies, together with our sex-risk factor indeterminate conclusion, underscore the current knowledge gaps on RVF risk factor mediation in humans. Our investigation of the relationship between household-level crowding and RVF seropositivity also revealed no association.

In addition, ethnicity was significantly associated with RVF seropositivity and the risk was highest among the Karimojong. One study in Kenya reported a high prevalence of RVF among the Turkana people who share an international border with the Karamojong.^51^ Both tribes are predominantly nomadic with a livelihood sustained by livestock, thus their occupational interaction with the animals could be responsible for their heightened risk to RVF. In earlier publications, data are scarce on the association between ethnicity and RVF seropositivity and this could be for three reasons. First, studies were conducted over small geographical areas occupied by the same ethnic group.^49^ Second, studies preferred to stratify on location or residence which could be far apart thus covering different ethnicities^51 52^ that are never delineated. In our study, despite the overlap between region of the country and ethnicity, region of the country was a weak predictor of RVF seropositivity and the association with ethnicity would have been missed if we had omitted ethnicity in our study design. Third, ethnicity has never been reported in relation to RVF such that scholars did not consider it as a potential predictor in their literature review and data collection. Much as we think this heightened risk in some ethnicities could be mediated through cultural practices related to greater animal–human interaction, the role of genetics should be considered in future studies.

This study contributes a portion to the generally sparse literature of RVF exposure burden in humans: a systematic review by Clark *et al* observed that only a third (34.5%) of RVF seroprevalence studies were conducted in humans, the rest in animals with 44.3% in livestock and 23% in wildlife.^39^ Our study provides insight on exposure to RVF in at-risk populations in Uganda, highlighting that outbreaks of severe disease occur against a background of unrecognised transmission. Second, we used a clustered survey design and included a large sample size characterised by 467 clusters and 1319 individuals. This enabled us to study risk factors such as household crowding that would otherwise not be investigated short of such a study design. We recommend that regular RVF seroprevalence studies should be conducted from the same population within endemic areas to monitor trends. To save on costs, these could be integrated in a timely manner within the well-funded national health surveys such as the HIV/AIDS indicator surveys, as was the case for our belated RVF seroprevalence study and UPHIA 2016/17 in Uganda. Studies that integrate the health of humans, animals and environment (‘One-health’) should concurrently be conducted in humans, domestic animals, poultry, wildlife and mosquitoes so as to investigate inter-species RVF transmission dynamics. These would be helpful in the early detection of emerging disease threats in animals before spilling over to humans, improving food safety and security, ensuring timely intervention and enhancing the currently loose multisectoral collaboration in surveillance and control.

Our study was limited by the inability to differentiate recent and long-established cases since only ELISA IgG tests were done but not IgM. The gold standard test for measuring RVF antibodies in plasma and serum is the use of virus neutralisation tests such as the plaque or focus reduction neutralisation assays. These tests require the use of live virus in a category 3 laboratory for which we did not have access to at the time. Therefore, our reported RVF seroprevalence could as well be biased, and the ‘true seroprevalence’ is either lower or higher. Second, we analysed stored plasma specimens from another study designed for a different purpose; hence, data on some potential risk factors of interest were not collected. In addition, the original UPHIA study was not powered to detect RVF antibody presence in humans, and our study was innately limited in its sample size as some districts had only 3–20 households included, thus unlikely to be representative of the population in those communities. The original UPHIA survey only included people aged 0–64 years. We believe this did not significantly affect our results as 97% of the Ugandan population is less than 65 years. Finally, only individuals in the cattle corridor are included. Inclusion of the population outside the cattle corridor would have provided good seroprevalence comparison, as the results we obtained could as well be the same prevalence outside the cattle corridor.

In conclusion, RVF infection is common among humans in Uganda’s cattle corridor and the exposure in humans is associated with animal and poultry ownership, as well as demographic attributes such as age and ethnicity. New ‘one-health’ RVF predictive, mitigation and control efforts should in addition to humans, animals and environment include poultry as potential neglected determinants of infection.

## Supplementary material

10.1136/bmjph-2025-002563 online supplemental file 1

## Data Availability

All data relevant to the study are included in the article or uploaded as supplementary information.
