# Supplementary material for: Rift Valley fever seroprevalence and risk factors among human populations in Uganda’s cattle corridor: a cross-sectional study
Source: BMJ Public Health. 2025 Jul 31;3(2):e002563. doi: 10.1136/bmjph-2025-002563 (PMC12315012; doi:10.1136/bmjph-2025-002563)
Supplement: online supplemental file 1 [file bmjph-3-2-s001.docx]

Supplementary Figure 1: RVF Seroprevalence by age and sex

Round or square blobs refers to the prevalence point estimates

Black vertical bars refers to the 95% confidence intervals

Supplementary Figure 2: Coefficient plot for the final level 3 multivariable mixed effects logistic regression analysis summarising the factors predictive of RVF seropositivity among humans in Uganda’s cattle corridor

Legend

Model 1: reference category for age group is “age group 0-24”; sex is “female”, and ethnicity is “Banyankore”

Model 2: reference category for education is “no formal education”, housing status is “corrugated iron sheets”, and country region is “central 1”

Model 3: reference category for water source is “underground/borehole”, residence is “urban”, own cattle is “own no cattle”; own sheep/goat is “own no sheep/goat”, own poultry is “own no poultry”

Vertical dotted line through 1 refers to point of no association

Round blobs and horizontal bars refers to the point estimates and 95% confidence intervals, respectively

Supplementary Table 1: Variables coding sheet

| **Variable** | **Variable description** | **Variable codes** |
| --- | --- | --- |
| ea_hhid_fixed | UPHIA survey household id | 4-digit unique household identification number |
| ea_hhid_ln_fixed | UPHIA survey participant id | 6-digit unique individual identification number |
| rvfseroepi_no_repos | RVF seroprevalence study id | 9-digit unique individual identification number |
| enrolledin_rvfepi | Eligibility status for RVF seroprevalence study | 0=No; 1=Yes |
| reasons_4_exclusion | Reason for ineligibility in RVF seroprevalence study | string |
| age | Age (years as continuous variable) | Numeric |
| agecut | Age group (MeSH age category) | 1=0-5; 2=6-12; 3=13-18; 4=19-24; 5=25-44; 6=45-64 |
| agecut2 | Age group (MeSH age category) | 1=0 – 24 years; 2=25 – 44 years; 3=45 – 64 years |
| sex2 | Sex | 0=Female; 1=Male |
| Ethnic3 | Ethnicity | 1=Banyankore; 2=Baganda; 3=Iteso; 4=Langi; 5=Karamojong; 96=Other ethnicity |
| UBOSREG10AIDSNAME | Region of the country | 1=Central 1; 2=Central 2; 3=Mid-North; 4=North-Eastern; 5=South-Western |
| DISTRICT | District | 1=Kiboga; 2=Luwero; 3=Mubende; 4=Nakasongola; 5=Rakai; 6=Ssembabule; 7=Lyantonde; 8=Mityana; 9=Nakaseke; 10=Kyankwanzi; 11=Katakwi; 12=Soroti; 13=Kaberamaido; 14=Amuria; 15=Serere; 16=Apac; 17=Kotido; 18=Lira; 19=Moroto; 20=Nakapiripirit; 21=Abim; 22=Amolatar; 23=Dokolo; 24=Kaabong; 25=Oyam; 26=Alebtong; 27=Amudat; 28=Kole; 29=Napak; 30=Otuke; 31=Mbarara; 32=Ntungamo; 33=Ibanda; 34=Isingiro; 35=Kiruhura |
| EducationLevel3 | Highest level of education attained | 1=No formal education; 2=Primary (P1-P7); 3=Secondary (S1-S6); 4=Tertiary (more than secondary) |
| ReligionCode3 | Religion | 1=Catholic; 2=Protestant/Anglican; 3=Muslim; 4=Other |
| Married3 | Marital status | 1=Never married; 2=Married or living together; 3=Separated (divorced/widowed) |
| MatRoof3 | Housing status (house roof material) | 1=Corrugated iron (mabati); 2=Thatch/palm leaf (makuti); 96=Other roof type |
| Pregnant2 | Currently pregnant | 0=No; 1=Yes; 96=n/a |
| hivstatusfinal | HIV status | 0=Negative; 1=Positive |
| hepb | Hepatitis B exposure | 0=Negative; 1=Positive |
| eversyphilis2 | Syphilis exposure | 0=Negative; 1=Positive |
| watersource3 | Water source | 1=Underground/borehole; 2=Piped water; 3=Surface water; 4=Other water sources |
| MOSNETS | Mosquito net use | 0=No; 1=Yes |
| rostercount | Crowding (household/cluster size)/ number of people in a household | 1=1-3; 2=4-6; 3=7-9; 4=10 or more |
| urban | Residence | 0=Urban; 1=Rural |
| OWNCOWS | Own cattle | 0=No; 1=Yes |
| OWNGTSHP | Own sheep/goat | 0=No; 1=Yes |
| OWNDOG | Own dog | 0=No; 1=Yes |
| OWNPLRY | Own poultry | 0=No; 1=Yes |
| OWNANML | Own other animals (camel, horse, donkey) | 0=No; 1=Yes |
| bloodspec_tested | Was plasma specimen tested for RVF virus antibodies | 0=No; 1=Yes |
| FinalEU | Final ELISA arbitrary units (AU) reading | Numeric value |
| rvf_statusfinal | Recorded RVF exposure status | 0=Negative; 1=Positive |

Supplementary Table 2: Assessing for collinearity among predictor variables selected for level 3 multivariable mixed effects logistic regression analysis

| Variable | Variable inflation factor (VIF) | Tolerance |
| --- | --- | --- |
| Age group | 1.05 | 0.96 |
| Sex | 1.06 | 0.94 |
| Ethnicity | 1.08 | 0.93 |
| Education level | 1.14 | 0.88 |
| Housing status | 1.03 | 0.97 |
| Region of the country | 1.09 | 0.92 |
| Water source | 1.03 | 0.97 |
| Residence | 1.12 | 0.90 |
| Own cattle | 1.26 | 0.79 |
| Own sheep/goat | 1.35 | 0.74 |
| Own poultry | 1.31 | 0.76 |
| Mean VIF 1.14 | | |

Supplementary Table 3: Assessing for correlation among predictor variables selected for level 3 multivariable mixed effects logistic regression analysis

| Variable | Age group | Sex | Ethnicity | Education level | Housing status | Region of the country | Water source | Residence | Own cattle | Own sheep/ goat | Own poultry |
| --- | --- | --- | --- | --- | --- | --- | --- | --- | --- | --- | --- |
| Age group | 1.0000 | - | - | - | - | - | - | - | - | - | - |
| Sex | 0.0044 | 1.0000 | - | - | - | - | - | - | - | - | - |
| Ethnicity | -0.0280 | -0.0092 | 1.0000 | - | - | - | - | - | - | - | - |
| Education level | -0.1908 | 0.2200 | -0.0030 | 1.0000 | - | - | - | - | - | - | - |
| Housing status | 0.0568 | -0.0082 | -0.0003 | -0.0258 | 1.0000 | - | - | - | - | - | - |
| Region of the country | 0.0289 | -0.0053 | -0.2061 | -0.0887 | 0.1264 | 1.0000 | - | - | - | - | - |
| Water source | -0.0207 | -0.0032 | 0.0486 | 0.0888 | -0.0236 | -0.1058 | 1.0000 | - | - | - | - |
| Residence | 0.0534 | 0.0368 | -0.0674 | -0.1519 | -0.0530 | 0.0813 | -0.0295 | 1.0000 | - | - | - |
| Own cattle | 0.0125 | 0.0619 | -0.1722 | -0.0083 | -0.0246 | 0.0798 | 0.0155 | 0.1411 | 1.0000 | - | - |
| Own sheep/goat | 0.0046 | 0.0397 | -0.0801 | -0.0071 | -0.0310 | 0.0878 | 0.0672 | 0.2197 | 0.3861 | 1.0000 | - |
| Own poultry | -0.0036 | 0.0455 | -0.0955 | -0.0181 | 0.0416 | 0.0280 | 0.0910 | 0.2239 | 0.3365 | 0.4171 | 1.0000 |

Supplementary Table 4: Assessing interaction between factors predictive of RVF seropositivity among humans in Uganda’s cattle corridor.

| Variables | aOR (95% CI) | Likelihood ratio test | |
| --- | --- | --- | --- |
|  |  | LR chi2 | p-value |
| **Age category and ethnicity** | | | |
| **Without interaction** | | 10.03 | 0.438 |
| 0 – 24 years | Reference (Ref) |  |  |
| 25 – 44 years | 2.77 (1.79 - 4.26) |  |  |
| 45 – 64 years | 3.01 (1.76 - 5.15) |  |  |
| Banyankore | Ref |  |  |
| Baganda | 1.80 (0.77 - 4.22) |  |  |
| Iteso | 2.59 (1.17 - 5.70) |  |  |
| Langi | 2.65 (1.23 - 5.73) |  |  |
| Karamojong | 3.54 (1.55 - 8.07) |  |  |
| Other ethnicity | 2.64 (1.18 - 5.89) |  |  |
| **With interaction** | |  |  |
| 0 – 24 years (Banyankore) | Ref |  |  |
| 25 – 44 years (Banyankore) | 8.25 (0.95 - 71.23) |  |  |
| 45 – 64 years (Banyankore) | 7.62 (0.75 - 77.06) |  |  |
| Banyankore (0 – 24 years) | Ref |  |  |
| Baganda (0 – 24 years) | 4.96 (0.58 - 42.81) |  |  |
| Iteso (0 – 24 years) | 3.67 (0.41 - 32.64) |  |  |
| Langi (0 – 24 years) | 7.78 (0.98 - 61.44) |  |  |
| Karamojong (0 – 24 years) | 10.81 (1.24 - 94.30) |  |  |
| Other ethnicity (0 – 24 years) | 6.17 (0.73 - 52.09) |  |  |
| 0 – 24 years X Banyankore | Ref |  |  |
| 25 – 44 years X Baganda | 0.20 (0.02 - 2.45) |  |  |
| 25 – 44 years X Iteso | 0.71 (0.06 - 7.77) |  |  |
| 25 – 44 years X Langi | 0.26 (0.03 - 2.60) |  |  |
| 25 – 44 years X Karamojong | 0.27 (0.02 - 2.97) |  |  |
| 25 – 44 years X Other ethnicity | 0.24 (0.02 - 2.59) |  |  |
| 0 – 24 years X Banyankore | Ref |  |  |
| 45 – 64 years X Baganda | 0.43 (0.03 - 6.10) |  |  |
| 45 – 64 years X Iteso | 0.55 (0.04 - 7.90) |  |  |
| 45 – 64 years X Langi | 0.22 (0.02 - 2.81) |  |  |
| 45 – 64 years X Karamojong | 0.17 (0.01 - 2.73) |  |  |
| 45 – 64 years X Other ethnicity | 0.76 (0.06 - 10.12) |  |  |
| **Age category and Owning cattle** | | | |
| **Without interaction** | | 1.32 | 0.516 |
| 0 – 24 years | Ref |  |  |
| 25 – 44 years | 2.86 (1.86 - 4.40) |  |  |
| 45 – 64 years | 2.84 (1.67 - 4.84) |  |  |
| No cattle | Ref |  |  |
| Own cattle | 2.04 (1.39 – 3.00) |  |  |
| **With interaction** | |  |  |
| 0 – 24 years (No cattle) | Ref |  |  |
| 25 – 44 years (No cattle) | 2.25 (1.21 - 4.21) |  |  |
| 45 – 64 years (No cattle) | 2.82 (1.31 - 6.04) |  |  |
| No cattle (0 – 24 years) | Ref |  |  |
| Own cattle (0 – 24 years) | 1.63 (0.83 - 3.21) |  |  |
| 0 – 24 years X No cattle | Ref |  |  |
| 25 – 44 years X Own cattle | 1.56 (0.66 - 3.67) |  |  |
| 45 – 64 years X Own cattle | 1.01 (0.35 - 2.92) |  |  |
| **Age category and Owning poultry** | | | |
| **Without interaction** | | 0.80 | 0.669 |
| 0 – 24 years | Ref |  |  |
| 25 – 44 years | 2.88 (1.87 - 4.44) |  |  |
| 45 – 64 years | 2.93 (1.72 – 5.00) |  |  |
| No poultry | Ref |  |  |
| Own poultry | 2.23 (1.42 - 3.50) |  |  |
| **With interaction** | |  |  |
| 0 – 24 years (No poultry) | Ref |  |  |
| 25 – 44 years (No poultry) | 2.28 (0.96 - 5.44) |  |  |
| 45 – 64 years (No poultry) | 1.87 (0.59 - 5.91) |  |  |
| No poultry (0 – 24 years) | Ref |  |  |
| Own poultry (0 – 24 years) | 1.71 (0.78 - 3.75) |  |  |
| 0 – 24 years X No poultry | Ref |  |  |
| 25 – 44 years X Own poultry | 1.36 (0.50 - 3.68) |  |  |
| 45 – 64 years X Own poultry | 1.78 (0.49 - 6.51) |  |  |
| **Ethnicity and Owning cattle** | | | |
| **Without interaction** | | 3.39 | 0.640 |
| Banyankore | Ref |  |  |
| Baganda | 1.60 (0.69 - 3.73) |  |  |
| Iteso | 1.94 (0.88 - 4.31) |  |  |
| Langi | 1.90 (0.88 - 4.12) |  |  |
| Karamojong | 3.08 (1.35 - 7.02) |  |  |
| Other ethnicity | 2.52 (1.14 - 5.60) |  |  |
| No cattle | Ref |  |  |
| Own cattle | 1.91 (1.27 - 2.88) |  |  |
| **With interaction** | |  |  |
| Banyankore (No cattle) | Ref |  |  |
| Baganda (No cattle) | 1.88 (0.66 - 5.36) |  |  |
| Iteso (No cattle) | 1.44 (0.44 - 4.74) |  |  |
| Langi (No cattle) | 2.64 (0.93 - 7.47) |  |  |
| Karamojong (No cattle) | 3.65 (1.21 - 11.07) |  |  |
| Other ethnicity (No cattle) | 2.33 (0.86 - 6.31) |  |  |
| No cattle (Banyankore) | Ref |  |  |
| Own cattle (Banyankore) | 2.22 (0.57 - 8.61) |  |  |
| Banyankore X No cattle | Ref |  |  |
| Baganda X Own cattle | 0.62 (0.10 - 3.65) |  |  |
| Iteso X Own cattle | 1.40 (0.26 - 7.47) |  |  |
| Langi X Own cattle | 0.57 (0.12 - 2.73) |  |  |
| Karamojong X Own cattle | 0.71 (0.13 - 3.74) |  |  |
| Other ethnicity X Own cattle | 1.33 (0.25 - 7.04) |  |  |
| **Ethnicity and Owning poultry** | | | |
| **Without interaction** | | 4.22 | 0.519 |
| Banyankore | Ref |  |  |
| Baganda | 1.52 (0.65 - 3.54) |  |  |
| Iteso | 2.00 (0.90 - 4.41) |  |  |
| Langi | 1.98 (0.92 - 4.28) |  |  |
| Karamojong | 3.43 (1.51 - 7.79) |  |  |
| Other ethnicity | 2.40 (1.08 - 5.35) |  |  |
| No poultry | Ref |  |  |
| Own poultry | 2.09 (1.32 - 3.32) |  |  |
| **With interaction** | |  |  |
| Banyankore (No poultry) | Ref |  |  |
| Baganda (No poultry) | 1.17 (0.28 - 4.99) |  |  |
| Iteso (No poultry) | 0.47 (0.049 - 4.42) |  |  |
| Langi (No poultry) | 1.95 (0.48 - 7.84) |  |  |
| Karamojong (No poultry) | 4.14 (1.14 - 15.04) |  |  |
| Other ethnicity (No poultry | 1.78 (0.49 - 6.47) |  |  |
| No poultry (Banyankore) | Ref |  |  |
| Own poultry (Banyankore) | 1.56 (0.41 - 5.92) |  |  |
| Banyankore X No poultry | Ref |  |  |
| Baganda X Own poultry | 1.49 (0.25 - 8.97) |  |  |
| Iteso X Own poultry | 5.37 (0.46 - 62.49) |  |  |
| Langi X Own poultry | 1.11 (0.20 - 6.01) |  |  |
| Karamojong X Own poultry | 0.77 (0.15 - 4.07) |  |  |
| Other ethnicity X Own poultry | 1.61 (0.31 - 8.33) |  |  |
| **Owning cattle and poultry** | | | |
| **Without interaction** | | 0.03 | 0.859 |
| No cattle | Ref |  |  |
| Own cattle | 1.71 (1.15 - 2.54) |  |  |
| No poultry | Ref |  |  |
| Yes poultry | 1.79 (1.13 - 2.86) |  |  |
| **With interaction** | |  |  |
| No cattle (No poultry) | Ref |  |  |
| Own cattle (No poultry) | 1.83 (0.78 - 4.32) |  |  |
| No poultry (No cattle) | Ref |  |  |
| Own poultry (No cattle) | 1.85 (1.04 - 3.30) |  |  |
| No cattle X No poultry | Ref |  |  |
| Own cattle X Own poultry | 0.92 (0.35 - 2.40) |  |  |
